# Supplementary material for: PLOS Genetics 2015 Reviewer Thank You
Source: PLoS Genet. 2016 Feb 23;12(2):e1005925. doi: 10.1371/journal.pgen.1005925 (PMC4764371; doi:10.1371/journal.pgen.1005925)
Supplement: S1 Reviewer List — (PDF) [file pgen.1005925.s001.pdf]

PLOS Genetics would like to thank all those who reviewed on behalf of the journal in 2015:

Alejandro Aballay
Mohamed Abdel-Rahman
Herman Aberle
John Abrams
Patrick Achard
Annita Achilleos
Martin Ackermann
Brian Ackley
David Adams
Keith Adams
Maja Adamska
Sankar Adhya
Simon Aeschbacher
Markus Affolter
Reuven Agami
Alexander Agoulnik
Nishant Agrawal
Aneil Agrawal
Hugo Aguilaniu
Andrés Aguilera
Ivan Ahel
Yashi Ahmed
Zubair Ahmed
Shawn Ahmed
Dag Ahren
Slimane Ait-Si-Ali
Elias Aizenman
Asifa Akhtar
Rosemary Akhurst
Bungo Akiyoshi
Anna Aksenova
Denise Al Alam
Eric Alani
M.Mar Alba
Sonja-Verena Albers
Frank Albert
Simon Alberti
David Albertini
Anders Albrechtsen
Joy Alcedo
Martí Aldea
Melinda Aldrich
Juan Alfonzo
Kari Alitalo
Ash Alizadeh
Ravi Allada
Rando Allikmets
Laura Almasy
Scott Alper
J. Andrew Alspaugh
Benjamin Altenhein
Philipp Altrock
José Álvarez-Castro
James Amatruda
Anna Amtmann
Wenfeng An
Bogi Andersen
Erik Andersen
James Anderson
Anne Anderson
Ryan Anderson
Magnus Andersson
Dan Andersson
Istvan Ando
Rose Andrew
Montserrat Anguera
Mireille Ansaldi
Aseem Ansari
Adam Antebi
Francesca Antonacci
Hana Antonicka
Christopher Antos
Takashi Aoyama
Suneel Apte
Charles Aquadro
Luís Aragón
Alexei Aravin
Jon Arch
José Argüello
Juan Lucas Argueso
John Aris
Robert Arkowitz
Brian Arnold
André-Patrick Arrigo
Masako Asahina
Atsushi Asakura
Miho Asaoka
Hosseinali Asgharian
Orr Ashenberg
Kaveh Ashrafi
Alan Ashworth
Stella Aslibekyan
Themistocles Assimes
Alan Attie
Liliana Attisano
Paul Auer
Yurii Aulchenko
Chantal Autexier
Tomer Avidor-Reiss
Philip Avner
Nagi Ayad
David Aylor
Julien Ayroles
Hela Azaiez
Erika Bach
Francois Bachand
Ekaterina Badaeva
Kristin Baetz
Michel Bagnat
Yong-Sun Bahn
Yuling Bai
Adam Bailis
Richard Baines
Duncan Baird
Julie Baker
Kristian Baker
Guus Bakkeren
Mohan Balasubramanian
Janneke Balk
David Baltrus
Leah Band
Yambazi Banda
Claudia Bank
Frederic Bantignies
Brent Bany
Fernando Baquero

Scott Baraban
Daniel Barbash
Allison Bardin
Menashe Bar-Eli
Naama Barkai
Alice Barkan
Scott Barolo
Maureen Barr
Luis Barreiro
Jeffrey Barrett
Antoni Barrientos
Rosa Barrio
Susan Baserga
Konrad Basler
Munira Basrai
George Bassel
Martine Bassilana
Albert Basson
Alexander Bassuk
Murat Bastepe
Boris Bastian
Uttiya Basu
Ken Batai
Joseph Bateman
Jacques Batut
L. Ryan Baugh
Ralf Baumeister
Isabel Bäurle
Simon Baxter
Vafa Bayat
Howard Baylis
Fuller Bazer
Christine Beattie
Terri Beaty
Mark Beaumont
Peter Becker
Esther Becker
Anke Becker
Antonio Bedalov
Bertrand Bed'Hom
Michael Beer
Martin Beer
Christian Beetz
Jonathan Beever
Gerrit Begemann
Jean Beggs
Thomas Begley
Jürgen Behrens
Richard Behringer
Traude Beilharz
Chase Beisel
Greg Beitel
Gill Bejerano
Alexandra Belayew
William Belden
Dominique Belin
Youssef Belkhadir
John Belknap
Jordana Bell
Douglas Bell
Stephen Bell
Xavier Belles
Daniel Belsky
Inna Belyantseva
Jennifer Benanti
Philip Benfey
Monsef Benkirane
Eva Benkova
Thomas Bennett
Eric Bennett
Jeffrey Bennetzen
Tamar Ben-Yosef
Michael Berenbrink
Jeremy Berg
Wolfgang Berger
Frederic Berger
Dave Berger
Alan Bergland
Andy Berglund
Dominique Bergmann
Ulfar Bergthorsson
Paul Berkman
Luiz Bermudez
Pascal Bernard
Andre Bernards
Thomas Bernhardt
Maud Bernoux
Kara Bernstein
Claire Bertet
Camille Berthelot
Lars Bertram
Joseph Besharse
Florence Besse
Mireille Betermier
Esther Betran
Jill Bettinger
Leo Beukeboom
Steven Bevan
Needhi Bhalla
Jahar Bhattacharya
Claude Bherer
Alessandro Bianchi
Alexander Bick
Sharon Bickel
Wendy Bickmore
Erhard Bieberich
Jason Bielas
Anja-Katrin Bielinsky
Leslie Biesecker
Stefano Biffo
Sue Biggins
Brad Binder
Albrecht Bindereif
Emanuele Biondi
James Birchler
Walter Birchmeier
Alexander Bird
C. William Birky, Jr.
Ewan Birney
Dorothy V. M. Bishop
Douglas Bishop
Ton Bisseling
Benoit Biteau
Trever Bivona
Brian Black
Peter Black
Stephen Blacklow
Craig Blackstone
T. Keith Blackwell
Seth Blair
William Blaner
Frank Blattner
Jesús Blázquez
John Blischak
Melanie Blokesch
Ian Blomfield
Jesse Bloom
Gareth Bloomfield
Elizabeth Blue
Justin Blumenstiel
Louis-Marie Bobay
Michael Boddy
Mikael Boden
Thomas Boehm

Arjen Boender
Daniel Bogenhagen
Laszlo Bogre
Magdalena Boguta
Astrid Böhne
Markus Bohnsack
Vilhelm Bohr
Ewelina Bolcun-Filas
Paul Bollyky
Michelle Bond
Joshua Bonkowsky
Carsten Bonneman
Warren Booth
John Boothroyd
Seth Bordenstein
Ingrid Borecki
Justin Borevitz
Anthony Borneman
G. Valentin Börner
Pavel Borodin
Alex Bortvin
Alexis Bosman
Yohan Bossé
Lionello Bossi
Vladimir Botchkarev
Eric Bouhassira
Cortney Bouldin
Simon Boulton
Rebecca Boumil
Deborah Bourc'His
Michael Boutros
Klaas Bouwmeester
Paola Bovolenta
Margot Bowen
Bruce Bowerman
Gregory Bowman
Adam Boyko
Jon Boyle
Yuri Bozzi
Benjamin Brachi
Jason Bragg
Clive Bramham
Marc Bramkamp
Michael Brandeis
Yaniv Brandvain
Dana Branzei
Gloria Brar
Robert Braun
Sarah Bray
Alexander Brehm
Björn Brembs
Chad Brenner
Emery Bresnick
Bonita Brewer
Miguel Brieno-Enriquez
Farren Briggs
Normand Brisson
Anne Britt
Kendal Broadie
Limor Broday
Peter Brodersen
Pnina Brodt
Steven Brody
Saverio Brogna
Heather Broihier
Samantha Brooks
Angela Brooks-Wilson
David Brow
Christopher Brown
Steven Brown
Grant Brown
Pamela Brown
Patrick Brown
Carolyn Brown
Katja Brückner
Benoit Bruneau
Anne Brunet
Vincent Bruno
Emme Bruns
Maja Bucan
Vanni Bucci
Martin Buck
Edward Buckler
Juan Bueren
Alex Buerkle
Stephen Buratowski
Emanuele Buratti
Lukas Burger
Boudewijn Burgering
Robert Burgess
Sean Burgess
Arthur Burghes
Gaetan Burgio
Daniel Burke
Laura Burrack
Lori Burrows
Vincent Burrus
William Bush
Kathryn Bushley
Michael Buszczak
Melinka Butenko
Samantha Butler
Geraldine Butler
Roger Butlin
Arthur Butt
Laura Buttitta
Mark Buttner
Peter Byers
Anders Bystrom
Javier Caceres
Jean Lud Cadet
James Cai
Qiuyin Cai
Huaibin Cai
Bradley Cairns
John Calarco
Kim Caldwell
George Calin
Patrick Callaerts
Judy Callis
Myriam Calonje
Mario Calus
James Calvet
Brian Calvi
Amy Camp
Daniel Campbell
Judith Campbell
Peter Campbell
Philippe Campeau
Nathalie Campo
Jose Cancelas
José Cansado
Xu Cao
Blanche Capel
Neil Caporaso
John Capra
Peter Carbonetto
Luis Cardenas
Maria Cardenas
Valerio Carelli
Karen Carleton
Gordon Carmichael
Agamemnon Carpousis
Antony Carr

Nicola Carraro
Sebastien Carreno
Ana Carrera
Russ Carstens
Dee Carter
Patrizia Casaccia
Nicholas Casewell
Tamara Caspary
Lynne Cassimeris
Irene Castano
Clare Casteel
James Castelli-Gair Hombría
Sergi Castellví-Bel
Diego Castrillon
Giacomo Cavalli
Eunyoung Chae
Li Chai
Yunrong Chai
Yang Chai
Sreekanth Chalasani
Ann Chambers
Severine Chambeyron
Danny Chamovitz
Raymond Chan
Danny Chan
Kin Chan
Jonah Chan
Guillaume Chanfreau
Hui-Yun Chang
Sandy Chang
Hsiao-Han Chang
Stephen Chanock
Michael Chao
Jocelyn Chapman
Clint Chapple
Marika Charalambous
Deborah Charlesworth
Brian Charlesworth
Frederic Charron
Larry Chasin
Nilanjan Chaterjee
Dipankar Chatterji
Dhruba Chattoraj
Taiping Chen
Xiao-Ya Chen
Zijiang Chen
Rui Chen
Jeremy Chen
Phang-Lang Chen
Z. Jeffrey Chen
Fang Chen
Di Chen
Meng Chen
Rong Chen
Han Chen
Bin Chen
Runsheng Chen
Qi Chen
Zhukuan Cheng
Ching-Yu Cheng
Louise Cheng
Soo-Chen Cheng
Chonghui Cheng
Yury Chernoff
Joshua Cherry
Louis Chesler
Zachary Cheviron
Fulvio Chiacchiera
Tsutomu Chiba
Peter Chien
Cheng-Ting Chien
Patrick Chinnery
Joanna Chiu
Chi-Chou Chiu
QueeLim Ch'ng
Keith Choe
Doil Choi
Kyunghee Choi
Woonyoung Choi
Kang Chong
Suyinn Chong
Ya-Hui Chou
Dipanjan Chowdhury
John Christie
Zofia Chrzanowska-Lightowlers
Mon-Li Chu
Chiou-Fen Chuang
George Chuck
Edward Chuong
Clemente Cillo
Elizabeth Cirulli
Brian Ciruna
Peter Claes
Thomas Clandinin
Leigh Clark
Andrew Clark
David Clark
Steven Clarke
Anne-Kathrin Classen
Ellen Clayton
John Cleary
Thomas Clemens
Thomas Cline
David Clouthier
David Cobrinik
J. Mark Cock
Enrico Coen
Paula Cohen
Barak Cohen
Ehud Cohen
Ronald Cohn
Mónica Colaiácovo
Stewart Cole
Francesca Cole
Martine Collart
Nansi Colley
Josep Comeron
Steve Conlan
Shannon Conley
Tim Connallon
Barbara Conradt
Miguel Constancia
Silvestro Conticello
Austin Cooney
Graham Coop
Timothy Cooper
Gregory Cooper
William Copeland
Samuel Coradetti
Kevin Corbett
Mark Corbett
Joseph Corbo
Heather Cordell
Dolores Corella
Seth Corey
Brendan Cormack
Davide Corona
Adrian Cortes
Peggy Cotter
James Cotterell
Justin Courcelle
Leah Cowen
Pamela Cowin

Michael Cowley
Jeffery Cox
Michael Cox
Nancy Cox
Jeffrey Craig
Lisa Craig
Erin Cram
Robert Cramer
Dana Crawford
Steve Crews
Julie Cridland
Gael Cristofari
Daniel Croll
John Cronan
Sean Crosson
Deborah Croteau
Nicole Crown
Györgyi Csankovszki
Chang-Yi Cui
Joan Curcio
Sean Curran
Patrick Curtis
Martha Cyert
Jack Da Silva
Xing Dai
Daniel Daley
Remus Dame
Alan D'Andrea
Richard Daneman
Weiwei Dang
Jeffery Dangl
Nadia Danilova
Nico Dantuma
Dawood Darbar
Sébastien Darras
Aparup Das
John Davey
Sean David
Lance Davidson
Irwin Davidson
Nicholas Davidson
Beverly Davidson
Shireen Davies
Nicholas G. Davis
Brigid Davis
Erica Davis
Igor Dawid
Brad Day
Elfride De Baere
Paul de Bakker
Mario de Bono
Marella de Bruijn
Jose F. de Celis
Dirk-Jan de Koning
Maria de la Torre
Stijn De Langhe
Nicholas De Lay
Gustavo de los Campos
Ruud de Maagd
Dirk de Rooij
Ive De Smet
Claudio De Virgilio
Caroline N. Dealy
Antony Dean
Michael Deans
Peter Dearden
Laurent Debarbieux
Jared Decker
Wim Declercq
Paola Defilippi
David Deitcher
Philip Dejager
Juan Carlos Del Pozo
Olivier Delaneau
Christos Delidakis
Florence Demenais
Fabio Demontis
Jeff Demuth
Tanneke Den Blaauwen
Marcel Den Hoed
Erick Denamur
Benjamin Deneen
Christophe D'Enfert
Wu-Min Deng
Megan Dennis
Elizabeth Dennis
Angela Depace
Bart Deplancke
Ludovic Deriano
Abby Dernburg
David Des Marais
Raymond Deshaies
Claude Desplan
Kevin Devine
Steve Devoto
Didier Devys
Andrew Dewan
Ajay Dhaka
Gill Diamond
Aaron Diantonio
Sam Díaz-Muñoz
Christine Didonato
Giorgio Dieci
Dennis Diener
Stephanie Diezmann
Brian Dilkes
Joseph Dillard
Patrick Dimario
Christian Dina
Mei Ding
Wen-Xing Ding
Jonathan Dinman
José Dinneny
Marc Dionne
Michael Dixon
Ron Do
Anna Dobritsa
Chris Doe
Paul Doetsch
Henrik Dohlman
Liam Dolan
Egor Dolzhenko
Anne Donaldson
Xinnian Dong
Aiwu Dong
Martin Donnelly
Nicole Donofrio
Mary Donohoe
Timothy Donohue
Gina Doody
Ben Dorshorst
Yali Dou
Jennifer Doudna
Angela Douglas
Simon Dove
John Dow
Steven Dowdy
James Dowling
Jessica Downs
Jeremy Draghi
David Draper
Gideon Dreyfuss
Monica Driscoll
Cord Drögemüller

Konstantinos Drosatos
Jacques Drouin
David Drubin
Iain Drummond
D. Allan Drummond
Daniela Drummond-Barbosa
Li-Lin Du
Dongsheng Duan
Wolfgang Dubiel
David Dubnau
Raymond Dubois
Edward Dubrovsky
Frank Dudbridge
Valerie Duffy
Michael Duffy
Nicolas Duforet-Frebourg
Yann Dufour
Priya Duggal
Guillaume Dumenil
Laramie Duncan
Bernard Duncker
Maitreya Dunham
Ian Dunham
Malcolm Dunlop
Ian Dunn
Julie Dunning Hotopp
Gary Dunny
Sally Dunwoodie
Richard Durbin
Robert Duronio
Troy Duster
Julien Dutheil
Aditya Dutta
Fabien Duveau
Jonathan Dworkin
Ian Dworkin
Kelly Dyer
Brian Dynlacht
Oliver Ebenhoeh
Johann Eberhart
Leo Eberl
Dieter Ebert
Arnaud Echard
Andrew Eckert
Winfried Edelmann
Bruce Edgar
Todd Edwards
Edward Egelman
Boris Egger
Patrick Eichenberger
Martin Eilers
David Eisenmann
Edward Eivers
Nels Elde
Olivier Elemento
Sarah Elgin
Peter M. Elias
Steve Elledge
Lisa Ellerby
Craig Ellermeier
Nathan Ellis
Steven Ellis
Ronald Ellis
Michael Elowitz
Maurice Elphick
Patrick Emery
Ben Emery
Kazuo Emoto
Astrid Engel
Nora Engel
Barbara Engelhardt
David Engelke
James Engert
Gregory Enns
Doug Epstein
Michael Epstein
Jeanette Erdmann
Sylvia Erhardt
Joachim Ernst
Lynda Erskine
Mafalda Escobar
Yuval Eshed
Jeffrey Essner
Mark Estelle
Todd Evans
John Ewer
Adam Ewing
James M. Fadool
William G. Fairbrother
Daniel Falush
Yun Fang
Charles Farber
Steven Farber
Hanna Fares
Michael Farkas
Cristiano Fava
Justin Fay
Eric Fearon
David Featherstone
Michael Federle
Ann Feeney
Robert Feil
Adam Feist
Laura Fejerman
Benjamin Feldman
Marie-Anne Félix
Jacques Fellay
Hanping Feng
Thomas Ferenci
David Ferguson
John Ferguson
Edwin Ferguson
Cristiano Ferlini
Oscar Fernandez-Capetillo
Javier Fernandez-Martinez
Alisdair Fernie
Aretha Fiebig
Maria Figueroa
Steven Finkel
Ruth Finkelstein
E. Jean Finnegan
Andreas Fischer
Richard Fishel
Simon Fisher
Elizabeth Fisher
Anna-Sophie Fiston-Lavier
Anthony Fiumera
Heather Fiumera
Jason Flannick
Thomas Flatt
Robert Fleischer
Jose Florez
Marco Foiani
Edan Foley
Lasse Folkersen
Pau Formosa-Jordan
Fabio Fornara
Mark Fortini
Kevin Foster
Gregory Fournier
John Fowler
Douglas Fowler
Claire Francastel
Michael Francis

Christopher Francklyn
Alison Frand
C. Andrew Frank
Laurent Frantz
Manfred Frasch
James Fraser
Timothy Frayling
Ian Frayling
Rachel Freathy
Merete Fredholm
Stephen Free
Michael Freeling
Michael Freitag
Catherine Freudenreich
Urban Friberg
Marc Friedlander
Alan Friedman
Timothy Friesen
Steven Frisch
Lars Fritsche
Marcus Fruttiger
James Fry
Bryan Fry
Andrew Fry
Judith Frydman
Xiang-Dong Fu
Guifang Fu
Robert Fuchs
Isabelle Fudal
Tatsuo Fukagawa
Ryuya Fukunaga
Simone Fulda
Tudor Fulga
Andre Furger
Eva Furrow
Masahiko Furutani
Bruce Futcher
Anthony Futerman
Toni Gabaldon
Daniel Gaffney
Oscar Gaggiotti
Dominique Gagliardi
George Gaitanis
Joshua Galanter
Rene Galindo
Peter Gallant
Angela Gallo
Jean Luc Gallois
Eric Gamazon
Li Gan
Fen-Biao Gao
L. Rene Garcia
Abel Garcia-Pino
David Gardner
David Garfield
David Garfinkel
Gian Garriga
Daniel Garrigan
Paul Garrity
Danielle Garsin
Marc Gartenberg
Anton Gartner
Jacob Garza
Anthony Garza
Audrey Gasch
Don Gash
Paolo Gasparini
Susan Gasser
Walter Gassmann
David Gatfield
Kyle Gaulton
Sergey Gavrilets
Simon Gayther
Maria Gazouli
Kai Ge
Brian Gebelein
Mary Gehring
Vincent Geli
Angie Gelli
Nicolas Gengler
Elisabeth Génot
André Gerber
Jeannine Gerhardt
Jennifer Gerton
Daniel Geschwind
Christopher Geyer
Pamela Geyer
Arjumand Ghazi
Mauro Giacca
Amato Giaccia
Greg Gibson
Gary Gibson
Carolyn Gibson
David Gifford
Clement Gilbert
Christian Gilissen
Sarjeet Gill
Kulvinder Gill
Matthew Gill
Peter Gillespie
Gabriele Gillessen-Kaesbach
Stephen Ginsberg
James Giovannoni
Tatiana Giraud
Santhosh Girirajan
Amy Gladfelter
Pierre Gladieux
Vadim Gladyshev
Christopher Glass
David Glass
Dominique Glauser
Jane Glazebrook
Thomas Glover
Matthew Goddard
Colin Goding
Lucy Godley
Hira Goel
Valérie Goguel
Omer Gokcumen
David Golan
Amy Goldberg
Andy Golden
Susan Golden
Anita Goldinger
Mary Goldring
Aaron Goldstrohm
Erica Golemis
Kent Golic
Mark Gomelsky
Edgar Gomes
Jose Luis Gomez-Skarmeta
Zachariah Gompert
Claudia Gonzaga-Jauregui
Cayetano Gonzalez
Josefa González
Acaimo González-Reyes
Gregory Goodall
Ursula Goodenough
John Goodier
Myron Goodman
Dmitry Gordenin
Christo Goridis
Harald Goring
Gregor Gorjanc

Boris Görke
Gohta Goshima
Toni Gossmann
Jonatha Gott
Joel Gottesfeld
Susan Gottesman
Alexander Gottschalk
Magdalena Götz
Fred Gould
Kathleen Gould
Mark Goulian
Geneviève Gourdon
Benjamin Gourion
Chhabi Govind
Susumu Goyama
Beata Grallert
Michael Granato
Murray Grant
Barth Grant
David Grattan
Jake Gratten
Peter Graumann
Simon Gravel
Stephen Gray
Daniel Gray
Joe Gray
Roger Greenberg
Casey Greene
Andy Greenfield
Ralph Greenspan
David Greenstein
Iva Greenwald
Celia Greenwood
Christopher Grefen
Richard Gregory
Brian Gregory
Stephen Gregory
Shiv Grewal
Courtney Griffin
Bernhard Grimm
Andrew Grimson
Alla Grishok
Alice Grob
Martien Groenen
Leopold Groesser
Leif Groop
Carol Gross
Susan Gross
Rudolf Grosschedl
Alan Grossman
Michael S. Grotewiel
Stephan Gruber
Elin Grundberg
Christopher Grunseich
Oliver Gruss
Nataly Grutenko
Sam Gu
Weifeng Gu
Yongtao Guan
Frederico Gueiros-Filho
Antoine Guichet
Tina Gumienny
Teresa Gunn
Kris Gunsalus
Hongwei Guo
Fang-Qing Guo
Yan Guo
Wangzhen Guo
Alexander Gusev
Patrice Guyenet
Jakob Haaber
Eric Haag
Steven Haase
James Haber
Jason Hackney
Stéphane Hacquard
Zena Hadjivasiliou
Suzana Hadjur
Christian Haering
Pejmun Haghighi
Matthew Hahn
Christopher Haiman
Sandra Hake
Randal Halfmann
Mario Halic
Martin Hallberg
Karen Halliday
Mary Halloran
Hiroshi Hamada
Yoko Hamazaki
Tina Hambuch
Geert Hamer
Bruce Hamilton
Sven Hammerschmidt
Thomas Hammond
Randy Y. Hampton
Yoo-Jeong Han
Hiroshi Handa
Christine Hann
Anthony Hannan
Wendy Hanna-Rose
Roberta Hannibal
Immo Hansen
Roger Hardie
Jeffrey Hardin
Wolf-Dietrich Hardt
Christian Hardtke
John Hardy
Tamar Harel
Vincent Harley
Michael Harms
Reuben Harris
Kelley Harris
Robert Harris
Steven Harris
Douglas A. Harrison
Lynn Harrison
Richard G. Harrison
Anne Hart
Anton Hartmann
Caroline Harwood
Paul Hasegawa
Hisashi Hashimoto
Bassem Hassan
Terry Hassold
Nicholas Hastie
Dolph Hatfield
Silke Hauf
Ronald Hause
Susanne Häussler
Thomas Hawn
Courtney Haycraft
John Hayes
Xin He
Xionglei He
Denis Headon
Edith Heard
Angela Heck
Olaf Heidenreich
Yael Heifetz
Lutz Hein
Matthias Heinig
Christian Heintzen
Erin Heinzen Cox

Carl-Philipp Heisenberg
James Hejtmancik
Siegfried Hekimi
Chris Helliwell
Gib Hemani
Ian Henderson
Rene Hendriksen
Steven Henikoff
Brenna Henn
Clarissa Henry
Yves Henry
Jennifer Herman
Gail Herman
Christophe Herman
Damien Hermand
Joachim Hermisson
Ryan Hernandez
Jussi A. Hernesniemi
Alfredo Herrera-Estrella
David Herrin
Ronna Hertzano
Hanspeter Herzel
Jane Hewitt
Veronica Heyningen
Meleah Hickman
Alicia Hidalgo
Peter Robin Hiesinger
Philip Hieter
Doug Higgs
Penelope Higgs
Friedhelm Hildebrandt
Caroline Hill
Geoffrey Hill
William Hill
Andreas Hiltbrunner
Gary Hime
Dirk Hincha
Yaniv Hinits
Veronica Hinman
Anke Hinney
Deborah Hinton
Jay Hinton
David Hipfner
Masami Y. Hirai
Candice Hirsch
Takashi Hishida
Christophe Hitte
Chris Hittinger
Thomas Hnasko
Katherine Hoadley
Robin Hobbs
Oliver Hobert
Asger Hobolth
James Hodge
Andrea Hodgins-Davis
Jan Hoeijmakers
Eva Hoffmann
Federico Hoffmann
Marten Hofker
Michael Hofreiter
Deborah Hogan
Paul Hohenlohe
Stefan Hohmann
Barbara Hohn
James Holaska
Steven Holland
Peter Holland
Andrew Hollenbach
Jill Hollenbach
Peter Hollenhorst
Nancy Hollingsworth
William Holloman
Edward Hollox
Hilma Holm
Scott Holmes
Edward Holmes
Sabine Hölter
Tessa Holyoake
Erika Holzbaur
Emily Holzinger
Gary Hon
Saul Honigberg
Timothy Hoover
Ian Hope
Thorsten Hoppe
Anita Hopper
Malcolm Horne
Sally Horne-Badovinac
Eran Hornstein
Valerie Horsley
Bernhard Horsthemke
Stefan Hortensteiner
Steve Horvath
Xingliang Hou
Steven Hou
Evelyn Houliston
Douglas Houston
Martin Howard
Jonathan Howard
Rachel Howard-Till
Alan Howe
Stephen Howell
Bryan Howie
Brooke Howitt
Jason Howitt
Niall Howlett
Barbara Howlett
Peggy Hsieh
Yen-Ping Hsueh
Yijuan Hu
Patrick Hu
Jian Hua
Jirong Huang
Tony Huang
Sui Huang
Xun Huang
Wen Huang
Aurélie Hua-Van
Simon Hubbard
Bernhard Hube
Armin Huber
Sariel Hubner
Meritxell Huch
Billy G. Hudson
Diarmaid Hughes
Robert Hughes
Stacie Hughes
Chi-Chung Hui
Jean Sebastien Hulot
Patricia Hunt
Craig Hunter
Neil Hunter
Douglas Huseby
Stefan Hüttelmaier
Anna Huttenlocher
Harald Hutter
Pirro Hysi
Antonio Iavarone
Michael Ibba
Alexander Idnurm
Leszek Ignatowicz
Taisen Iguchi
Koichi Iijima
Akihiro Ikeda

Hae Kyung Im
Yuzuru Imai
Erik Ingelsson
Gwyneth Ingram
Roger Innes
Masayori Inouye
Carlo Iomini
Ivan Iossifov
Grzegorz Ira
Alan Irvine
Norio Ishida
Genichiro Ishii
Hiroshi Iwasaki
Robert Jackman
Scott Jackson
Francoise Jacob-Dubuisson
Hamed Jafar-Nejad
Andrew Jaffe
Euan James
Timothy James
Guilhem Janbon
Florence Janody
Michael Jantch
Andrew Jarman
Artur Jarmolowski
Heinrich Jasper
Jean-Paul Javerzat
Patrick Jay
James B. Jaynes
Albert Jeltsch
Eric Jenczewski
Jeffrey Jensen
Choongwon Jeong
Jukka Jernvall
Rolf Jessberger
Jenna Jewell
Songtao Jia
Liwen Jiang
Huaqi Jiang
Rulang Jiang
Duo Jiang
Yuling Jiao
Chris Jiggins
Peng Jin
Marek Jindra
Sue Jinks-Robertson
Hanna Johannesson
Kristen Johansen
Uwe John
Carl Johnson
Arlen Johnson
Erik Johnson
Tracy Johnson
F. Bradley Johnson
Andrew Johnson
Philip Johnson
Louise Johnson
Laura Johnston
David Jones
Kevin Jones
Louise Jones
Thomas Jongens
Marcel Jonkman
Philip Jordan
Caroline Josefsson
Raja Jothi
Laurent Journot
Marko Jovanovic
Gabor Juhasz
Kirsten Jung
Heinz Jungbluth
Peter Juo
Ivan Juric
Melissa Jurica
David Kadosh
Daniel Kaganovich
Jon Kaguni
Toshie Kai
Bernd Kaina
Angela Kaindl
Theodosia Kalfa
Isgouhi Kaloshian
Auinash Kalsotra
Naoki Kanayama
Nolan Kane
Sang Won Kang
Phillipp Kapranov
Jaakko Kaprio
Katrin Karbstein
François Karch
Gabrielle Kardon
Ted Karginov
Michael Karin
Robert Karn
Durgadas P. Kasbekar
Yona Kassir
Judith Kassis
Norihiro Kato
Richard Katz
Aris Katzourakis
Jay Kaufman
Masakado Kawata
Alex Kazantsev
Scott Keeney
Paul Keim
Erin Kelleher
Beat Keller
Manolis Kellis
William Kelly
Jeffrey Kelly
Hughes Kelly
Gavin Kelsey
Robert Kelsh
Kenneth Kendler
Scott Kennedy
Peter Kennelly
Linda Kenney
Juha Kere
Andrew Kern
Tom Kerppola
Benedikt Kessler
Rene Ketting
Johannes Kettunen
Philipp Khaitovich
Ahmad Khalil
Hemant Khanna
Mustafa Khokha
Rama Khokha
Chiea Khor
Alexander Khoruts
Kiyoshi Kikuchi
Patricia Kiley
Sunghoon Kim
Dennis Kim
John Kim
Hongkyun Kim
Tae-Hee Kim
Minsu Kim
Minjung Kim
Joomyeong Kim
Dong Wook Kim
William Kim
Akinori Kimura
Andreas Kindmark

Tami Kingsbury
John Kirby
Laura Kirkman
Antonis Kirmizis
Matias Kirst
Krzysztof Kiryluk
Jun Kitano
Robert Kittel
Avihu Klar
Nancy Kleckner
Kenneth Kleene
Gary Kleiger
Bruce Klein
Hannah Klein
Ophir Klein
Robert Kleta
Scott Klewer
Daniel Kliebenstein
Kimberly Kline
Michele Klingbeil
Larry Klobutcher
Wigard Kloosterman
Gabriele Klug
Marc Knight
Michael Knop
Joshua Knowles
Hirokazu Kobayashi
Tatsuya Kobayashi
Hans-Georg Koch
Ingrid Kockum
Daniel Koenig
Andrew Koff
Gou-Young Koh
Isaac Kohane
Kathryn Kohl
Annegret Kohler
Claudia Köhler
Suneil Koliwad
Richard Kolodner
Masaaki Komatsu
Sek Won Kong
James Konopka
Bon-Kyoung Koo
Michael Koob
Maarteen Koorneef
R. Frank Kooy
Artyom Kopp
Amnon Koren
Thomas Kornberg
Kerry Kornfeld
Daniel Kornitzer
Vyacheslav Korshunov
Ron Korstanje
Hendrik Korswagen
Arthur Korte
Martin Korte
Sergei Kosakovsky Pond
Noora Kotaja
Ákos Kovács
Rhett Kovall
Lukasz Kozubowski
Peter Kraft
Deborah Krakow
Igor Kramnik
David Krantz
Michael Krause
Henry Krause
Ronald Krauss
Rachel Kraut
Martin Kreitman
Michael Kristensen
Christopher Kristich
Daniel Kronauer
Marcus Kronforst
Arthur Kruckeberg
Sergey Kryazhimskiy
Damian Krysan
Ulrich Kück
Grzegorz Kudla
Jason Kuehner
Ashok Kulkarni
Jerzy K. Kulski
Carol Kumamoto
Rajiv Kumar
Justin Kumar
Shoen Kume
Gary Kupfer
Martin Kupiec
Hiroki Kurihara
Mitzi Kuroda
Hitoshi Kurumizaka
Teymuras Kurzchalia
Zoltán Kutalik
Tatiana Kutateladze
Andrei Kuzminov
Vladimir Kuznetsov
David Kwiatkowski
Michael Kyba
Bruno Kyewski
Charalambos Kyriacou
Michel Labouesse
Soni Lacefield
Raj Ladher
Michael Laessig
Robert Lahue
Marikki Laiho
Diana Laird
Robert Lake
Jean Charles Lambert
Sarah Lambert
David Lambeth
Michael Lampson
Robert Landick
Joseph Landry
Christian Landry
David Lane
B. Franz Lang
Douglas Langbehn
Thomas Langer
Charles Langley
Peter Lansdorp
Chiara Lanzuolo
Tuuli Lappalainen
Bret Larget
Luis Larrondo
Erica Larschan
Erik Larsson
Jan Larsson
Janine Lasalle
Paul Lasko
Florent Lassalle
Brittany Lasseigne
Michael Lassig
Wyndham Lathem
Nelson Lau
Vincent Laudet
Laura Lavine
Heather Lawson
Losif Lazaridis
Beth Lazazzera
Brian Lazzaro
Roland Le Borgne
Laurent Le Cam
Arnaud Le Rouzic

David Leach
Judith Leatherman
Matthew Lebo
Marc-Henri Lebrun
Eric Lecuyer
Seung-Jae Lee
Min Gyu Lee
Yong-Hwan Lee
Myung-Shik Lee
Jongmin Lee
Siu Sylvia Lee
Grace Lee
Cheng-Ruei Lee
Kyu-Sun Lee
Sang Eun Lee
Changhan Lee
Chi-Hon Lee
Tosso Leeb
Peter Leegwater
Véronique Lefebvre
Michael Leffak
Jean-Luc Legras
Gaelle Legube
Michael Lehmann
Ulrich Lehmann
Ben Lehner
Christian Lehner
Elissa Lei
Sergey Leikin
Birgit Leitinger
Bruno Lemaitre
Sylvain Lemeille
Johannes R. Lemke
Michael Lenhard
Jay Lennon
Pierre Léopold
Thierry Lepage
Emmanuelle Lerat
Inna Lermontova
Pascale Lesage
Christina Leslie
Elizabeth Leslie
Michelle Letarte
Laura Lettice
Jun-Yi Leu
Gerhard Leubner
François Leulier
Mitchell Paul Levesque
Bruce Levin
Tera Levin
Edward Levine
Michael Levine
Jean-Pierre Levraud
Zachary Lewis
Peter Lewis
Joshua Lewis
Brian A. Lewis
Klaus Ley
Ottoline Leyser
Ming Li
Xin Li
Chuanyou Li
Liming Li
Xin Li
Willis Li
Yi Li
Jianming Li
Ning Li
Shaoguang Li
Jia Li
Hongzhe Li
Jun Li
Bibo Li
Chris Li
Han Liang
Liming Liang
Hong Liao
Romain Libbrecht
Domenico Libri
Peter Lichter
Andrew Lidral
Susan Liebman
Mary Lilly
Janghoo Lim
Xiaorong Lin
Fucheng Lin
Huawen Lin
Rueyling Lin
Su-Ju Lin
Shuo Lin
Chentao Lin
Lasse Lindahl
Annika Lindblom
Gabriella Lindgren
Jiqiang Ling
Brian Link
Christoph Lippert
Mathew Littlejohn
Liang Liu
Yule Liu
Xigang Liu
Haoping Liu
Yi Liu
Yuan Liu
Hong-Xiang Liu
Hong Liu
Yie Liu
Chunqiao Liu
YaoGuang Liu
Dajiang Liu
Yifan Liu
Andy LiWang
Ana Llopart
Xavier Llor
Bertrand Llorente
Kirill Lobachev
John Logsdon
Po-Ru Loh
Dietmar Lohmann
Kirk Lohmueller
Konrad Lohse
Nicholas J. Loman
Fanxin Long
Sharon Long
Michelle Longworth
Luliana Lonita-Laza
Ruth Loos
Joseph Loparo
Javier López
Daniel López
Miguel López
Luis Lopez-Molina
Javier Lopez-Rios
Benjamin Loppin
Michael Lorenz
Alexander Lorenz
Katie E. Lotterhos
Huiqiang Lou
Paul Love
Kate Loveland
John Lovell
Robin Lovell-Badge
David Low
Hua Lu

Hua Lu
Pingli Lu
Qing Lu
Yuan Lu
Xiaowei Lu
Jining Lu
Francesca Luca
Reini Luco
Bryan Luikart
Ben Luisi
Vicki Lundblad
Erik Lundquist
David Lunt
Joe Lutkenhaus
David Lydall
Jens Lykke-Andersen
Kristen Lynch
Christopher Lynch
Vincent Lynch
Amy Lyndaker
Liang Ma
Li Ma
Hong Ma
Wenbo Ma
Danielle Maatouk
Paul Macdonald
Clinton C. MacDonald
Todd Macfarlan
Lee Machado
James MacLean
Lesley MacNeil
Amy MacQueen
Frank Madeo
Morris Maduro
Tatsuya Maeda
Dixie Mager
Erna Magnusdottir
Moe R. Mahjoub
Julio Maia
Martin Maiden
Berenike Maier
Gabi Maimon
Paul Mains
Domenico Maiorano
William Mair
Amit Majithia
Ho Yi Mak
Tak Mak
Christopher Makaroff
Hisaji Maki
Svetlana Makovets
Fransiska Malfait
Pal Maliga
Harmit Malik
Snezana Maljevic
Anna Malkova
Danielle Malo
John Malone
Julin Maloof
J. Robert Manak
Pablo Manavella
Eugenio Mancera
Lolitika Mandal
Ani Manichaikul
Judith Mank
James Manley
Suzanne Mansour
Chiara Manzini
Jessica Mar
Richard Maraia
Daniel Marenda
William Margolin
Morasso Maria
Urko Marigorta
Elizabeth Marin
John Maris
Debora Marks
Michael Marks
Eirini Marouli
Luciano Marraffini
Carmen Marsit
Adele Marston
Jurgen Marteijn
Kelsey Martin
Paul Martin
James Martin
Maureen Martin
Laetitia Martin
Alicia Martin
Christa Martin
Maria Dolores Martin-Bermudo
José Martínez
Alfonso Martinez-Arias
Jean-Claude Martinou
Keri Martinowich
Stefano Marzi
Joanna Masel
Daniel Masison
Ruth Massey
Catherine Masson-Boivin
Tatsuru Masuda
Hisao Masukata
Peter Mathers
Iain Mathieson
Ivan Matic
Ichiro Matsumura
Sachihiro Matsunaga
Makoto Matsuoka
Manuel Mattheissen
Mark Mattoson
David Matus
Mikhail Matz
Martin Matzuk
Cedric Maurange
Matthew Maurano
Satyajit Mayor
Thibault Mayor
Kimberly McCall
John McCarrey
Joseph McCarty
David McClay
Kathleen McCoy
Richard McCulloch
Laurence McCullough
Michael McEachern
Suzanne McGaugh
Philip McGinnity
Kelly McGowan
Patrick McGrath
Alistair McGregor
Bruce McKee
Kim McKim
Brett McKinney
Paul McLaren
Irwin McLean
Joel McManus
Michael McMurry
Elizabeth McNally
Allan McRaei
Gavin McStay
Mitch McVey
Graham McVicker
Rene Medema

Jeffrey Medin
Kay Medina
Monica Medina
Richard Meehan
Heather Mefford
Paul Megee
Colin Meiklejohn
Miriam Meisler
Peter Meister
Michael Meisterernst
Victoria Meller
Barbara Mellone
Karyn Meltz Steinberg
Stephen Meltzer
Stephen Melville
Raphaël Mercier
Houra Merrikh
Alexey Merz
Ludwine Messiaen
Ravikanth Metlapally
Brian Metzger
Mark Metzstein
Diogo Meyer
Ralph Meyer
Justin Meyer
Joel Meyer
David Meyre
Matthew Michael
Grégoire Michaux
Bénédicte Michel
Gracjan Michlewski
Christian Mielke
Alan Mighell
Tâm Mignot
Marco Milan
Christine Milcarek
Dusanka Milenkovic
Alistair Miles
Sarah Millar
Sean Millard
W. Todd Miller
Michael Miller
Craig Miller
Dana Miller
Daniel Miller
Bruce Miller
David Miller Iii
Kevin Mills
Tohru Minamino
Alex Minella
Baruch Minke
Mario Mirisola
Marie Mirouze
Christen Mirth
Dan Mishmar
Neeti Mishra
Hari Misra
David Mitchell
Aaron Mitchell
Phil Mitchell
Thomas Mitchell
Ryo Miyazaki
William Moar
Danesh Moazed
Kenneth Moberg
Nadja Møbjerg
Naoki Mochizuki
Axel Mogk
Karen Mohlke
Timothy Mohun
Calvin Mok
Antoine Molaro
Isabella Moll
Darren Monckton
Kelly Monk
David Monk
Raymond Monnat, Jr.
Anne-Helene Monsoro-Burq
Jacques Montagne
Martin Montecino
Courtney Montgomery
Stephen Montgomery
Silvia Monticelli
Kristi Montooth
Lieve Moons
Sean Moore
Darren Moore
Priya Moorjani
Carlos Moraes
Aixa Morales
Charles Moran
John Moran
Nancy Moran
Kevin Morano
Javier Morante
Robert Morell
Roy Morello
Eduardo Moreno
Celine Morey
Phil Morgan
David Morgan
Yasu Morita
Yoshinori Moriyama
Katherine Morley
Alexandre Morozov
David Morris
Patrick Morrison
Joachim Morschhäuser
James Moseley
Rebecca Mosher
Tom Moss
Richard Mott
Joanna Mountain
M. Maral Mouradian
Zissimos Mourelatos
Bernard Moussian
Loukas Moutsianas
Bryan Moyers
Iva Mozgova
Marek Mraz
Syed Mubarak Hussain
Gloria Muday
Gary Muehlbauer
Srabani Mukherjee
M. Shahid Mukhtar
Jürg Müller
Stefan Müller
John Mulley
Marcus R. Munafo
John Mundy
Silvia Munoz-Descalzo
José Muñoz-Dorado
Alysson Muotri
Elizabeth Murchison
Coleen Murphy
Peter Murray
Andrew Murray
Heath Murray
Joseph Murray
John Murray
Ben Murrell
Kiran Musunuru
Josyf Mychaleckyj
Chad Myers

Martin Myers
Sua Myong
Michael Myre
Max Nachury
Hanspeter Naegeli
Bhushan Nagar
Valakunja Nagaraja
Emi Nagoshi
Laszlo Nagy
Ferenc Nagy
Angus Nairn
Sonia Najjar
Hidewaki Nakagawa
Toru Nakamura
Yuki Nakamura
Keiichi Nakayama
Jun-Ichi Nakayama
Eiji Nambara
Jeremy Nance
Andre Nantel
Vagheesh Narasimhan
Iñigo Narvaiza
Dick R. Nässel
William Navarre
Nicolas Navarro
Andreas Nebenführ
Serge Nef
Mark Neff
Nicolas Negre
Richard Neher
John Neidhardt
James Neil
Joel Neilson
Christoffer Nellåker
Peter Nelson
Matthew Nelson
Christian Neri
Karla Neugebauer
Ekkehard Neuhaus
Jeff Neul
Ralph Neumüller
Phillip Newmark
Matilda Newton
Huu Nguyen
Frank Nicholas
Garth Nicholson
Dan Nicolae
Alain Nicolas
Michael Niederweis
Carien Niessen
Isao Nishimura
Patsy Nishina
Lee Niswander
Michael Nitabach
John Nitiss
Ryusuke Niwa
Dean Nizetic
Suzanne Noble
Justin Nodwell
Sumihare Noji
Kazuya Nomura
Daniel Nomura
Ken-Ichi Nonomura
Mohamed Noor
Fernando Noriega
Hope Northrup
Mariusz Nowacki
Romana Nowak
Noa Noy
Masafumi Nozawa
Vardis Ntoukakis
Evgeny Nudler
Leta Nutt
Sergey Nuzhdin
Todd Nystul
Rebecca Oakey
Jon Oatley
Darren Obbard
Carole Ober
Elke Ober
John D. Oberdick
Thomas O'Brien
Lucy O'Brien
David O'Brochta
Moira O'Bryan
Howard Ochman
Matthew O'Connell
Timothy O'Connor
Michael O'Connor
Michael O'Donnell
Marlene Oeffinger
Patrick O'Farrell
Yuya Ogawa
Bermseok Oh
Kevin Ohlemiller
Andreas Ohlmann
Robin Ohm
Yukinori Okada
Giles Oldroyd
Megan Oliva
Wendy Olivas
Raquel Oliveira
Antonio Oliver
Brian Oliver
Dominik Oliver
Richard Oliver
Timothy Olson
David Olson
James Olzmann
Rachel O'Neill
László Orbán
Richard Ordway
David Ornitz
Eyleen O'Rourke
Neil Osheroff
Oren Ostersetzer-Biran
Elaine Ostrander
Hong Ouyang
Tom Owen-Hughes
Edward Owusu-Ansah
Ertugrul Ozbudak
Annalise Paaby
Andrea Page-McCaw
Athma Pai
Ken Paigen
Kenneth Paigen
Päivi Pajukanta
Csaba Pal
Pier Francesco Palamara
Javier Palatnik
James Palis
Mark Pallen
Jianwei Pan
MingXin Pan
Junmin Pan
Tao Pan
Udai Pandey
John Panepinto
Michael Pankratz
Barbara Panning
Anne Paoletti
Manolis Papamichos-
Chronakis
Kai Papenfort

Fernando Pardo-Manuel De Villena
Leslie Parent
Christian Parisod
Hee-Moon Park
Christian Parker
Colin Parrish
John Parsch
Will Parsons
Linda Partridge
Janet Partridge
Leopold Parts
Bogdan Pasaniuc
Philippe Pasero
Ketan Patel
Chirag Patel
Marianna Patrauchan
Ned Patterson
Hubert Pausch
Pavlos Pavlidis
Youri Pavlov
Wojciech Pawlowski
Joshua Payne
Elspeth Payne
Francois Payre
Bret Payseur
Raha Pazoki
Gregory Pazour
Christopher Pearson
David Pederson
Thoru Pederson
Itsik Pe'Er
Michiel Pegtel
Mark Peifer
Stephan Peischl
Vladimir Pelicic
Laurence Pelletier
Gina Peloso
Paivi Peltomaki
Trevor Pemberton
Miguel Penalva
Sarah Pendergrass
Christopher Penfold
Jamy Peng
Melissa Pepling
Gislene Pereira
Minoli Perera
Brian Perkins
Michael Perlin
Nicole Perna
John Perry
George Perry
Matthias Peter
Morten Petersen
Christian Petersen
Svend Petersen-Mahrt
Thomas Petes
Marie-Agnès Petit
Enrico Petretto
John Petrini
Dmitri Petrov
Jonathan Pettitt
Roberto Pezza
Marcus Pezzolesi
Boris Pfander
Nitin Phadnis
Catherine Phelan
Patrick Phillips
Franck Pichaud
Curtis Pickering
Alison Pidoux
Craig Pikaard
Ramesh Pillai
Yitzhak Pilpel
Manuel Piñeiro
Lionel Pintard
Roger Pique-Regi
Matti Pirinen
Chrysoula Pitsouli
Antonius Plagge
Serge Plaza
Jeffrey Pleiss
Anne Plessis
Jonathan Plett
Stefanie Pöggeler
Joe Pogliano
Kit Pogliano
Christian Pohl
Laurent Poirel
Norbert Polacek
Martin Pollak
David Poller
Andrew Pomiankowski
Emilie Pondeville
Philip Poole
David Popham
Steven Porter
Douglas Portman
Daniel Portnoy
György Pósfai
Kenneth Poss
John Postlethwait
Alex Postma
Judith Potashkin
Wayne Potts
Joseph Powell
Robert Power
Ted Powers
Siddharth Prakash
Supriya Prasanth
James Prendergast
Mary Preuss
Jeffrey Price
Alkes Price
Clive Price
Michael Prigge
Victoria Prince
Richard Proia
Nick Proudfoot
Stephen Proulx
Sergi Puig
Patricia Pukkila
Jonathan Puritz
Zachary Pursell
Xiaoquan Qi
Ling Qi
Feng Qiao
Genji Qin
Huan Qiu
Luis Quadri
Christine Queitsch
Thomas Quertermous
Christopher Quince
Marcel Quint
Lluis Quintana-Murci
Isabella Quinti
Leonard Rabinow
Joshua Rabinowitz
Oliver Rackham
Cristina Rada
Rosa Rademakers
Laurel Raftery
Padinjat Raghu
Mosur Raghuraman

Abdul Hakkim Rahamathullah
David Raible
Tracy Raivio
David Raizen
Towfique Raj
Indika Rajapakse
Vardhman Rakyan
Sohini Ramachandran
Kumaran Ramamurthi
Mani Ramaswami
Amanda Ramos
Dale Ramsden
Oliver Rando
David Rasmussen
Morten Rasmussen
John Rathjen
Rajiv Rattan
Tommer Ravid
Anandasankar Ray
Soumya Raychaudhuri
Yevgeniy Raynes
Francisco Real
Mark Rebeiz
Mario Recker
Prabhakara Reddi
Peter Reddien
Françoise Redini
Christophe Redon
Laura Reed
Michael Reese
Birgitte Regenberg
Andreas Reichert
Josephine Reinhardt
Tânia Reis
Bing Ren
Rainer Renkawitz
Stefan A. Rensing
Francis Repoila
Antonio Reverter
Todd Reynolds
Nick Rhind
Paulo Ribeiro
Stephen Richards
Eric Richards
J. Brent Richards
Kristy Richards
Klaus Richter
Joel Richter
Daniel Rico
Lynn Riddiford
Christopher Ridout
Arne Rietsch
Karel Riha
Jason Rihel
Bruce Riley
Sylvie Rimsky
Vera Rinaldi
Diego Rincon-Limas
Maurice Ringuette
Samuli Ripatti
Makarand Risbud
Jens Rister
Marylyn Ritchie
Francois Robert
Richard Roberts
Peter Robinson
Gene Robinson
Elise Robinson
Sonia Rocha
Christian Rocheleau
Matthew Rockman
Pedro Rodriguez
Laura Rodriguez
Enrique Rodriguez-Boulan
Thomas Roeder
Henry Roehl
Igor Rogozin
Rori Rohlfs
Ignasi Roig
Melissa Rolls
Pascale Romby
Christopher Rongo
Stéphane Ronsseray
Dennis Roop
Marilyn Roossinck
Randall Roper
Michael Rosbash
Ann Rose
Vicki Rosen
Ben Ross
Derrick Rossi
Jeffrey Ross-Ibarra
Siegfried Roth
Scott Rothbart
Lawrence Rothblum
Adrian Rothenfluh
Joel Rothman
François Rouyer
David Rowitch
Peter Roy
Jason Rudd
David Rudner
Christian Rudolph
Elena Rugarli
Davide Ruggero
Natividad Ruiz
Andres Ruiz-Linares
Daniel Runcie
Steven Russell
Guy Rutter
Ilya Ruvinsky
Joske Ruytinx
Kathleen Ryan
Robert Ryan
Ivan Rychlik
Paolo Sabelli
Alessandra Sacco
Matthew Sachs
Kirsten Sadler Edepli
Takashi Sado
Jeroen Saeij
Alvaro Sagasti
Isabelle Sagot
Goutam Sahana
Yusuke Saijo
Shigeaki Saitoh
Takuya Sakaguchi
Isaac Salazar-Ciudad
Julian Sale
Iris Salecker
Carla Saleh
Maya Saleh
Muneeb Salie
Torbjörn Säll
Veikko Salomaa
Kaitlin Samocha
Maurilio Sampaolesi
Aravinthan Samuel
David Samuelson
Yolanda Sanchez
Suzanne Sandmeyer
Tzu-Kang Sang
Rafael Sanjuan

Vijay Sankaran
Serena Sanna
Manuel Santos
Dos Sarbassov
Vittorio Sartorelli
Paolo Sassone-Corsi
David Sassoon
Peter Satir
Makoto Sato
Ken Sato
Masamitsu Sato
Leonor Saúde
Sophie Saunier
Sophie Saunier
Isabel Saur
Sharon Savage
David Savage
Cathy Savage-Dunn
Sigal Savaldi-Goldstein
Dan Savic
Barry Saville
Outi Savolainen
Hidetoshi Saze
Peter Scacheri
Suzie Scales
Aylwyn Scally
Roel Schaaper
Joseph Schacherer
Beat Schaefer
Matthias Schaefer
Frank Schaeffel
Patrick Schafer
Christopher Schardl
Manfred Schartl
David Schatz
Marc Schaub
Konrad Scheffler
Eyal Schejter
Alexander F. Schier
Stephan Schiffels
Mario Schiffer
Philipp Schiffer
Carl Schildkraut
John Schimenti
Karen Schindler
Ernestina Schipani
Christine Schlacher
Patrick Schloss
Christian Schlötterer
Paul Schmidt
Laura Schmidt
Miriam Schmidts
Robert Schmitz
Monika Schmoll
Ralf Schneggenburger
David Schneider
David Schneider
Kay Schneitz
Jonathan Schoenecker
Mark Schöttler
Joshua Schraiber
Daniel Schrider
Julian Schroeder
Dirk Schübeler
Edward Schuchman
Markus Schuelke
Klaus Schughart
Rebecca Schüle
Hinrich Schulenburg
Robert Schulz
Molly Schumer
Heribert Schunkert
Trudi Schupbach
Christoph Schuster
Martin Schwaerzel
Tanja Schwander
Yuri Schwartz
François Schweisguth
Marilia Scliar
Douglas Scofield
William Scott
Rodney Scott
Donald Scott
Patrick Seale
Jonathan Sebat
James Seeb
Kimberley Seed
Ole Seehausen
Nava Segev
Cecile Segonzac
Michael Sehorn
H. Steven Seifert
Sonia Sen
Yasin Senbabaoglu
Cathal Seoighe
Tricia Serio
Karen Sermon
Aswin Seshasayee
Federico Sesti
Carmine Settembre
Laurent Seugnet
Veronika Sexl
Jagesh Shah
Premal Shah
Shai Shaham
Yousif Shamoo
John Shannon
Michael Shapira
Cynthia Sharma
Dror Sharon
Andrew Sharp
Phillip Sharp
Paul Shaw
Polina Shcherbakova
Michael Sheehan
Xia Shen
Binghui Shen
Wen Shen
Jason Shepherd
Dean Sheppard
Gavin Sherlock
Michael Sherman
William Sherwin
Xiaobing Shi
Yongshen Shi
Darryl Shibata
Takehiko Shibata
Alexandra Shields
Sagiv Shifman
Sebastian Shimeld
Osamu Shimmi
Alexander Shingleton
Tetsuro Shinoda
Akira Shinohara
Janet Shipley
Dorothy Shippen
Ken Shirasu
Patrick Shiu
Alena Shkumatava
David Shore
Daniel Shriner
Mark Shriver
Leah Shriver
Hui-Kuo Shu

Stanislav Shvartsman
Julia Sidorova
Ellen Sidransky
Mark Siegal
Kellee Siegfried
Sarah Siegrist
Stephan Sigrist
Robert Silverman
Amanda Simcox
Rosalia Simmen
Lyle Simmons
Matthew Simon
Martine Simonelig
Matias Simons
Kai Simons
Yuval Simons
Tatum Simonson
Gordon Simpson
Joe Leigh Simpson
Don Sin
Richard Sinden
Harinder Singh
Nadia Singh
Saurabh Sinha
Himanshu Sinha
Haruhiko Siomi
Mikiko Siomi
Linda Siracusa
Anand Sitaram
Daniel Skelly
Michael Skinner
Pontus Skoglund
Jane Skok
Jan Skotheim
Efthimios Skoulakis
Frank Slack
Robert Slany
Jon Slate
Matthew Slattery
Meghan Slean
Jason Slot
R. Keith Slotkin
Michel Slotman
Sebastien Smallwood
Craig Smibert
Desmond Smith
Andrew Smith
Gerald Smith
Wanli Smith
Lee Smith
Alicia Smith
Susan Smith
William Smith
Lisa Smith
Rachel Smith-Bolton
Ron Smits
Marcus Smolka
William Snell
Emilie Snell-Rood
R. Elizabeth Sockett
Kee Hoon Sohn
Nick Sokol
Isabel Sola
Jordi Solana
Jürgen Soll
Matthias Soller
Suzanne Sommer
Zhou Songyang
Wim Soppe
Rotem Sorek
Wayne Sossin
Martha Soto
Andrea Sottoriva
Alexandra Soukup
Rita Sousa-Nunes
Tim Sparwasser
Joseph Spatafora
Doug Speed
Terence Speed
Malte Spielmann
Charles Spillane
Pietro Spitali
Steven Spoel
Simon Sprecher
Janet Sprent
Martin Srayko
Shankar Srinivas
Supriya Srinivasan
Jagan Srinivasan
Raymond J. St. Leger
Peter Stambrook
Stefan Stamm
Konstantina Stankovic
Pamela Stanley
Peter Stanton
Jeremy Stark
Michelle Starz-Gaiano
Mike Steel
Oliver Stegle
Michael Steinbaugh
Walter Steiner
Eirikur Steingrimsson
Ulrich Stelzl
Derek Stemple
Deborah Stenkamp
Ioannis Stergiopoulos
Paul Sternberg
Ruth Steward
Douglas Stewart
James Stewart
A. Francis Stewart
Rodney Stewart
Bangyan Stiles
Michael Stitzel
Hugo Stocker
David Stone
Mark Stoneking
John Storey
Gisela Storz
Lucia Strader
Brian Strahl
Aaron Straight
Michael Strand
Phyllis Strauss
Joseph Strauss
Andrea Streit
Samuel Strom
Lena Ström
Beth Stronach
Lisa Strug
Lisa Stubbs
Eva Stukenbrock
Jörg Stülke
Francoise Stutz
Tin Tin Su
Tatiana Subhkhankulova
Maria Sueli Felipe
Michihiko Sugimoto
Greg Suh
Yousin Suh
Karsten Suhre
Patrick Sulem
Zhaoxia Sun
Lei Sun

Jianjun Sun
Meng-Xiang Sun
Xingmin Sun
Patrick Sung
Z. Renee Sung
Ida Surakka
Michael Surette
Beat Suter
Mark Sutton
Tsutomu Suzuki
Toru Suzuki
Petr Svoboda
Billie Swalla
Jeremy Swann
Maurice Swanson
Michael Swarbrick
Joann Sweasy
Luc Swevers
Bryan Swingle
Lorraine Symington
Moriah Szpara
Bruce Tabashnik
Kikue Tachibana-Konwalski
Jimmy Tai
Minoru Takata
Makoto Taketo
Toru Takumi
William Talbot
John Tamkun
Patrick Tan
Patrick Tan
Katsunori Tanaka
Shuuitsu Tanaka
Kayoko Tanaka
Motomasa Tanaka
Guy Tanentzapf
Zhengzheng Tang
Wei-Hua Tang
Hiromu Tanimoto
Milos Tanurdzic
Nicolas Tapon
Rachael Tarlinton
Kei Tashiro
Stefan Taubert
Diethard Tautz
Arianna Tavanti
Simon Tavare
Paulo Tavares
Carla Taveggia
Dan Tawfik
Hugh Taylor
Bruce Taylor
Robert Taylor
Sarah Teichmann
Luis Teixeira
Maria Teresa Teixeira
Mustafa Tekin
Aurelio Teleman
Liesbet Temmerman
Peter Ten Dijke
Shu-Chun Teng
Jason Tennessen
Yik-Ying Teo
Ron Tepper
Andrew Teschendorff
Jens Tetens
Maria Teves
Joshua Thaler
Martin Thanbichler
Ioannis Theodorou
Ulrich Theopold
Pascal Therond
William Theurkauf
Johan Thevelein
Dennis Thiele
Duncan Thomas
Jainy Thomas
Rachael Thomas
Stefan Thor
David Thorburn
Jeremy Thorner
Kevin Thornton
Randal Tibbetts
Irene Tiemann-Boege
Hwei-Fang Tien
Lubov Timchenko
Vincent Timmerman
Stephen Ting
Marc Tischkowitz
Sarah Tishkoff
Heidi Tissenbaum
David Tobin
David Toczyski
Nobuhiko Tokuriki
David Tollervey
Seth Tomchik
Alan Tomkinson
Ian Tomlinson
Navtej Toor
Laszlo Tora
Nicolás Toro
Attila Toth
Melissa Toups
Aminata Toure
Jeffrey Townsend
Paul Trainor
Elizabeth Tran
Lam-Son Tran
Arne Traulsen
Todd Treangen
Nathan Treff
Mathias Treier
Kimberly Tremblay
Jeffrey Trent
Eckardt Treuter
William Trimble
James Truman
Stephen Tsang
Christian Tschudi
Miltos Tsiantis
Bryan Tsou
Stephen Kwok-Wing Tsui
Hironaka Tsukagoshi
Susan Tsunoda
Shinobu Tsuzuki
Abraham Tucker
Anders Tunlid
Kürsad Turgay
B. Gillian Turgeon
Thomas Turner
Jens Tyedmers
Yehuda Tzfati
Masaru Ueno
Henriette Uhlenhaut
Helle Ulrich
James Umen
Robert Unckless
Mark Ungerer
Klaus Unsicker
Chris Upton
Nathaniel Urban
Makoto Urushitani
Nicole Valenzuela
Richard Vallee

Tom van Agtmael
Sander van den heuvel
Esther van der Knaap
Alexander van der Linden
Jan van der Meer
George van der Merwe
Mark Van Doren
Dik van Gent
Veronica van Heyningen
Ambro van Hoof
Jeremy Van Raamsdonk
Kristel Van Steen
Bas van Steensel
David Van Vactor
Klaas van Wijk
Russell Vance
Juan Vaquerizas
Jean-Pierre Vartanian
Daniel Vasiliauskas
Karen Vasquez
Shobha Vasudevan
Shashaank Vattikuti
Timothy Vaughan
Cyrus Vaziri
Jan-Willem Veening
Jan Veenstra
Victor Velculescu
Andre Verdel
Kristen Verhey
Esther Verheyen
Marie-Hélène Verlhac
Kevin Verstrepen
Monika Vetter
Chad Vezina
Beatriz Vicoso
Silvia Vidal
Marc Vidal
K. VijayRaghavan
Usha Vijayraghavan
Jordi Vila
Bjarni Vilhjalmsson
Diego Villar
Anne Villenueve
David Vincent
Patrick Viollier
Karen Visick
Rosella Visintin
Veronique Vitart
Charles Vite
Kerstin Voelz
Thierry Voet
Christine Vogel
Jean-Nicolas Volff
Pelin Cayirlioglu Volkan
Erik Vollbrecht
Tobias von der Haar
Thomas Vondriska
Michiel Vos
Rome Voulhoux
Andreas Wachter
Scott Waddell
Elmar Wahle
James Wakefield
Fergal Waldron
David Walker
Amy Walker
Graham Walker
Daniel Walker
Daniel Wall
Peggy Wallace
Chris Wallace
John Wallingford
Timothy Walsh
Jens Walter
Dirk Walther
Lucas Waltzer
Nancy Walworth
Jianmin Wan
Haibin Wang
Wen Wang
Xuemin Wang
Chung-Ju Wang
Jian Wang
Yue Wang
Ping Wang
Xinnan Wang
Xuelu Wang
Zhao-Wen Wang
Meng Wang
Kai Wang
Zhiyong Wang
Biao Wang
Zuoheng Wang
Xindan Wang
Jia-Wei Wang
Jue Wang
P. Jeremy Wang
Jianbo Wang
Elizabeth Want
Matthew Warman
Maria Warnefors
Jonathan Warner
Jens Waschke
M. Todd Washington
Katja Wassmann
Claus Wasternack
Yoshinori Watanabe
Chris Waters
Christine Watson
Jennifer Watts
Michael Weale
David Weaver
Ashley Webb
Michael Weber
David Weetman
Daniel Wegmann
Michael Wegner
Grzegorz Wegrzyn
Bin Wei
Xiangyun Wei
Gilbert Weidinger
Dolf Weijers
Marc Wein
Seth Weinberg
Robert Weinberg
Alan Weiner
Ted Weinert
John Weinstein
William Weis
Eric Weiss
Lauren Weiss
Mitchell Weiss
Louis Weiss
Daniel Weissman
Matthew Weitzman
Ronald Wek
Carrie Welch
John Welch
Kathryn Wellen
Raymund Wellinger
Michael Welte
Xiaoquan Wen
Jurgen Wendland
Kerstin Wendt

Joel Wertheim
Monte Westerfield
Chris Westlake
Lynn Westphal
Harm-Jan Westra
Kristi Wharton
Rob White
Benjamin White
Patricia White
Michael White
Charles White
Bradley White
Malcolm Whiteway
Tanya Whitfield
Chris Whitfield
Alexander Whitworth
Hynek Wichterle
Thomas Wicker
Claude Wicker-Thomas
Reed Wickner
Stefanie Widder
Andrzej Wierzbicki
Sadie Wignall
Julia Wilflingseder
Claus Wilke
Jon Wilkins
Simon Williams
Robert Williams
Thomas Williams
James Williamson
Ian Willis
Felix Willmund
James Wilson
James F. Wilson
Michael Wilson
Samuel Wilson
Megan Wilson
Daniel Wilson
Clive Wilson
Thomas Wilson
Melissa Wilson Sayres
Tim Wiltshire
Jeffrey Wilusz
Verena Wimmer
Mark Winey
Rebecca Wingert
Malcolm Winkler
Wade Winkler
Beate Winner
Fred Winston
Roger Wise
Stephan Witt
Curt Wittenberg
James Wohlschlegel
Fred Wolf
Yuri Wolf
Kenneth Wolfe
Sungho Won
Kwong-Kwok Wong
Alex Wong
Jason Wong
Roger Woodgate
John Woolford
Jerry Workman
Naomi Wray
Margaret Wrensch
Stephen Wright
Xifeng Wu
C.-Ting (Ting) Wu
Michael Wu
Jian-Qiu Wu
Zhihao Wu
Thomas Wynn
Anthony Wynshaw-Boris
Rongwen Xi
Sheng Xiao
Xinshu Xiao
Daoxin Xie
Li Xin
Jian Xu
Lin Xu
Tongda Xu
Xiang-Min Xu
Jin-Rong Xu
Lei Xue
Ramin Yadegari
Gen Yamada
Yumi Yamaguchi-Kabata
Shinya Yamamoto
Tadashi Yamamoto
Kotaro Yamamoto
Kenshi Yamasaki
Yukiko Yamashita
Masakazu Yamazaki
Wei Yan
José Yáñez
Ming Yang
Shuhua Yang
Ivana Yang
Xiangli Yang
Shi-Bing Yang
Judith Yanowitz
Xuebiao Yao
Ayse Yarali
Christopher Yau
Chun Ye
Bing Ye
Yihong Ye
Gene Yeo
Rui Yi
Soojin Yi
Eda Yildirim
Yanhai Yin
Jerry Yin
Timothy York
Kohta Yoshida
Furuta Yoshikazu
Douglas Young
Elton Young
Fengwei Yu
Hongtao Yu
Zhongsheng Yu
Bin Yu
Jianming Yu
Jae-Hyuk Yu
Michael Yudell
Katherine Yutzey
Jean-François Zagury
Ronen Zaidel-Bar
Noah Zaitlen
Jolanta Zakrzewska-Czerwinska
Luis Zaman
Sarah Zanders
David Zappulla
David Zarkower
Mihaela Zavolan
Martin Zeidler
Susanne Zeilinger
Andrew Zelhof
Robert Zeller
Elazar Zelzer
Kai Zeng
Magdalena Zernicka-Goetz

Xiuren Zhang
Chuan-Xi Zhang
Jinghui Zhang
Jin-Song Zhang
Yong-Zhen Zhang
Yong Zhang
Zhenguo Zhang
Guojie Zhang
Xianlong Zhang
Xiaolan Zhang
Yu Zhang
Kang Zhang
Xiaoyu Zhang
Xian Sheng Zhang
Yunde Zhao
Jing Zhao
Li Zhao
Xiaolan Zhao
Mei Zhen
Binglian Zheng
Siyuan Zheng
Degui Zhi
Xiaotian Zhong
Rui Zhou
Jiliang Zhou
Yun Zhou
Zhemin Zhou
Xiang Zhou
Yihua Zhou
Heng Zhu
Jinsong Zhu
Denise Zickler
Ewa Zietkiewicz
Stephanie Zimmerman
Robert Zimmermann
Kai Zinn
Sebastian Zoellner
Brian Zoltowski
Aldert Zomer
Fei Zou
James Zou
Yimin Zou
Stephan Zuchner
Jianru Zuo
Jian Zuo
